# Transactions of the Medical Society of the State of New York

**Published:** 1851-06

**Authors:** 


*Transactions of the Medical Society of the State of New-York, during its Annual Session, held at Albany, February 4th,* 1851.

The Transactions of the State Society for 1851, make an octavo of two hundred and forty-eight pages. They are published as one of the documents of the Assembly, at the public expense, and are therefore, we presume, in the hands of most of the members of the medical profession in this State. Several interesting papers are contained in the volume. The address by the late President, Dr. Alexander Thompson, is a sensible, well written production. With this the volume opens. The second article is entitled, "Observations on some points in Medical Ethics, by John McCall, M. D." We regret to find in this article a personal attack upon a practitioner residing at the same place with the author, and a distinguished member of the profession of this State — an attack, as it seems to us, hardly consistent with that ethical tone which it is the design of the article to inculcate. The following extract contains the charge to which we refer:

"It has often occurred to me, that some of our practitioners in the country, and now and then one or more of the distinguished professors of our schools of medicine in the cities, have without due reflection given certificates and recommendations in favor of a particular medicinal compound, or instrument, or contrivance of some sort or other as being thought by somebody altogether better than any thing of the kind in use.

To illustrate my meaning, I will mention a case. While I had the honor of being president of this society, I was called upon at my office in the morning, while the social and kindly feelings of our nature are usually more accessible and accommodating, by a Mrs. B., who wished to obtain a recommendation for her abdominal and uterine supporters, stating that she had heard of my official and professional standing, and therefore desired a certificate from the president of the medical society of the State of New-York, in order that her very useful articles of trade might thus become better known, and sooner introduced to the notice of the profession and the public.

Here was a case, as I saw at once, in which no ordinary share of firmness and independence would be needed, and fortunately both came to my aid.

In personal appearance, she was rather elegant and good looking, well formed and well dressed, with a goodly stock of jewels about her neck and fingers; and in addition to these attractions she possessed fine conversational powers, as far as could be judged of from the statement made respecting the superior qualities of the things she wished me to recommend. Of course she was received and treated with all the attention and respect to which a lady is entitled. But as to giving a certificate or recommendation to her or any body else, for such purpose, was entirely out of the question; and this resolution was frankly stated to her.

She appeared evidently disappointed, and manifested indications of ill feeling, and with an air of triumph and exultation, showed me certificates she had just obtained from two of the brotherhood of our city, and what was mortifying, was the fact that one of them was a professor in one of the medical schools of our city."

Now, as it is well known who is the professor residing at Utica, and as there is but one professor in a medical school who does reside in that city, the allusion can be regarded in no other light than a pointed, personal attack. Would this be justifiable, assuming that the person assailed had, apparently, committed a breach of medical ethics? We trow not: members of the medical profession should, to say the least, be not less charitable than the law requires; and, in law, an individual is presumed to be innocent of an offence until he is proved guilty. If Dr. McCall had charges to prefer against a fellow practititioner in his own city, the proper course is pointed out in the code of medical ethics adopted by the society to which his article was submitted. But we are very far from conceding, except for argument's sake, that, in the fact mentioned, there was any breach of medical ethics whatever. If professor Coventry regarded the "abdominal uterine supporters," contrived by "Mrs. B.," as useful instruments, it was perfectly proper for him to recommend them: nay, it was his duty so to do. The case is widely different from one in which a recommendation of a secret nostrum is obtained. There is no parallel between the two instances. This seems to us so perfectly obvious, that it would be a waste of time to argue the point. Dr. McCall, it would appear, has adopted a rule not to give certificates of approval of any invention or improvement. It does by no means follow, however, that Dr. Coventry is bound by the same rule; and if Dr. Coventry looks upon the matter in a different light from Dr. McCall, it certainly will not be conceded by the friends of the former, that he, is on that account necessarily wrong! We cannot but think the passage here quoted was written hastily, and that the impropriety of the personality, and the groundlessness of the imputation will be as apparent to the author, on reflection, as we are sure it must be to every reader.*

As the colleague and friend of Professor Coventry, we may be permitted to add, that there is not in the ranks of the profession of this, or any other country, a gentleman more deeply imbued with a high sense of honor, and a jealous regard for the character of the profession.

Article *third*, is on "Institution for the instruction of the deaf and dumb." By P. VanBuren, M. D.

Article *fourth*, "Address before the Medical Society of Oneida county." By M. M. Bagg, M. D.

Article *fifth*, "Abstract of cases from case-book," by John McCall, M. D.

Article *sixth*, is entitled "Homœopathy illustrated." By Thomas W. Blatchford, M. D. This is reprinted from the Transactions of 1843. It is a long article, occupying seventy pages. The able author considers elaborately, comprehensively, and satisfactorily, the merits of the homœopathic delusion.

Article *seventh*, is "An address delivered before the Rensselaer county Medical Society." By Thomas C. Brinsmade, M. D., President. Subject — The Medical Topography of Troy, N. Y.

Article *eighth*, is "A prize dissertation on the pernicious influences of nostrums or secret remedies upon the morals and health of the community." By John G. Sewell, M. D., of New-York. The prize of $20 was offered by Prof. Clark, of New-York. Seven essays were offered.

Article *ninth*, is devoted to notices of deceased members. Mr. Luther Guiteau, M. D., of Oneida county; Dr. Charles Babcock, of Oneida county; and Lester Green, M. D., of Herkimer county.

We have contented ourselves with thus giving a list of the articles, inasmuch as copies of the transactions will doubtless be received by a large portion of our readers. They contain gratifying evidences of the prosperity and usefulness of the society. Its advantages however, would be increased by an augmented interest, leading to a larger supply of contributions, from which to select articles for publication, and to a fuller representation of the profession of the State, at its regular meetings. The new plan of holding semi-annual meetings in different places will, we trust, conduce to a better appreciation of the importance of a State organization than now obtains.

It will be borne in mind that the semi-annual meeting of the State Medical Society will be held in Buffalo, on the second Tuesday, in the present month, June tenth.

---

* Since this was written, a certificate of recommendation of certain articles of trade, namely "Tilden & Co.'s Pure Medicinal Extracts," given by the very society to which Dr. McCall's communication was submitted, has met our eye. It may be seen in connection with Tilden & Co.'s advertisements in the advertising sheet of this Journal, given under the hand of the Secretary of the N. Y. State Society.

